# Surgical Correction of Pulmonary Stenosis Using Beating‐Heart Cardiopulmonary Bypass in a Dog With Persistent Left Cranial Vena Cava

**DOI:** 10.1155/crve/6139084

**Published:** 2026-04-17

**Authors:** Shuji Suzuki, Sachiyo Tanaka, Nobuo Kanno, Takuya Yogo, Yasuji Harada, Yasushi Hara

**Affiliations:** ^1^ Laboratory of Veterinary Surgery, Nippon Veterinary and Life Science University, Musashino, Tokyo, Japan, nvlu.ac.jp

## Abstract

A 7‐month‐old female toy poodle was referred for corrective surgery of pulmonary stenosis with persistent left cranial vena cava (PLCVC). Thoracic radiographs showed right heart enlargement. Echocardiography revealed moderate right ventricular hypertrophy and severe pulmonic stenosis with PLCVC. Right ventricular outflow tract reconstruction of severe pulmonary artery stenosis was performed under cardiopulmonary bypass. Intraoperative ventricular fibrillation occurred and was successfully treated with defibrillation; no postoperative complications were appreciated at the 1‐month recheck. At the 6‐month follow‐up, the dog was in good general condition without exercise intolerance, and an echocardiogram revealed that the pulmonary artery velocity at the narrow area had decreased. This case suggests that surgical correction using cardiopulmonary bypass is a feasible treatment option for severe pulmonary stenosis with PLCVC, particularly in small dogs in which catheter‐based intervention is technically challenging.

## 1. Introduction

Pulmonary stenosis (PS) is one of the most common congenital cardiac diseases in dogs and is characterized by obstruction of right ventricular outflow, leading to pressure overload of the right ventricle [1–3]. Balloon valvuloplasty is generally considered as the first‐line treatment for valvular PS because it is less invasive and has been associated with favorable clinical outcomes in many cases [4, 5]. Persistent left cranial vena cava (PLCVC) is a rare congenital anomaly resulting from the failure of the left cranial cardinal vein to regress during fetal development. Instead, it persists and typically drains into the coronary sinus, subsequently entering the right atrium [6]. The prevalence of PLCVC in dogs with congenital heart disease is reported to be 0.8%–4.5% [3, 7]. Although PLCVC alone rarely causes clinically significant hemodynamic compromise, it can complicate cardiac surgery by altering venous return pathways and obscuring critical anatomical structures, particularly during procedures such as patent ductus arteriosus (PDA) ligation or surgery for vascular ring anomalies [8, 9]. In dogs with severe PS complicated by PLCVC, selection of an optimal therapeutic approach can be challenging, particularly in small patients, due to limitations in catheter‐based intervention and complex vascular anatomy [10, 11]. In such cases, surgical correction under cardiopulmonary bypass may represent an alternative option when catheter‐based intervention is not suitable. This report describes the clinical findings, surgical management, and outcome of a dog with severe PS complicated by PLCVC that was successfully treated using beating‐heart cardiopulmonary bypass.

## 2. Case Presentation

A 7‐month‐old female toy poodle weighing 2.5 kg was referred to the Veterinary Teaching Hospital at Nippon Veterinary and Life Science University for evaluation of heart murmur. The dog showed mild pallor of the mucous membranes and exercise intolerance at the time of the first visit.

Auscultation revealed a Grade V/VI systolic ejection murmur with the point of maximum intensity at the left heart base. Complete blood count and serum chemistry panel results were within normal limits. Thoracic radiographs with right lateral and ventrodorsal projections revealed right atrial and ventricular enlargement and a bulge in the area of the pulmonary artery. The vertebral heart score (VHS) was 12.1, which was above the generally accepted upper limit of normal for dogs (10.5) [12]. The ECG revealed sinus rhythm with right axis deviation (MEA, −104°) and a deep S wave in Lead II (2.0 mV), supporting right ventricular enlargement. Two‐dimensional echocardiography showed the presence of moderate right ventricular concentric hypertrophy and moderate thickening of the pulmonary valve. The main pulmonary artery was dilated (Figure 1a,b). The pulmonary annulus diameter was almost the same compared with the aortic annulus: pulmonary annulus (10.6 mm); aortic annulus (10.6 mm) (Figure 1c). Mild systolic doming and commissural fusion of the pulmonic valve were also observed. Collectively, these findings were most compatible with a type A valvular PS (Video S1). The Doppler examination showed a severe stenotic gradient (maximum velocity [PV max] of 5.45 m/s; maximum systolic pressure gradient of 118.81 mmHg) (Figure 1d). The mean systolic pressure gradient across the pulmonary valve (PV mean gradient), obtained by tracing the continuous‐wave Doppler envelope, was 76.39 mmHg. The velocity time integral (VTI) across the aortic valve (VTI_AV) was 10.1 cm, the VTI across the pulmonary valve (VTI_PV) was 93.48 cm, and the VTI ratio (VTI_AV/VTI_PV) was 0.108.

Figure 1Echocardiographic images of right parasternal short‐axis views of the heart on initial examination. (a, b) Right parasternal long axis view of the pulmonary artery showed the thickening of the pulmonary valve and stenosis. (c) Aortic and pulmonary annulus diameters. Pulmonary artery hypoplasia was not observed. (d) Transthoracic continuous wave Doppler echocardiography of the right ventricular outflow tract. The systolic velocity across the stenosis is 5.45 m/s, and the pressure gradient is 119 mmHg according to Bernoulli equation.

**Figure:**
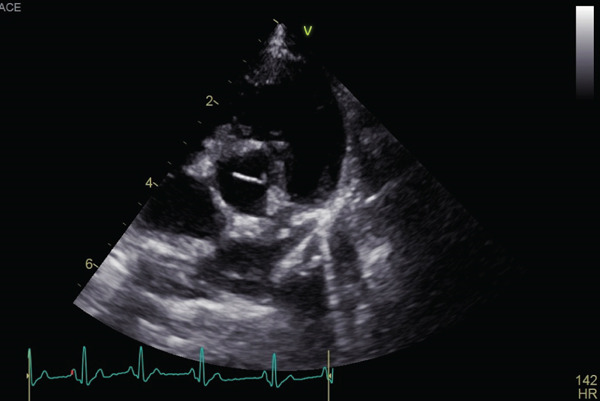
(a)

**Figure:**
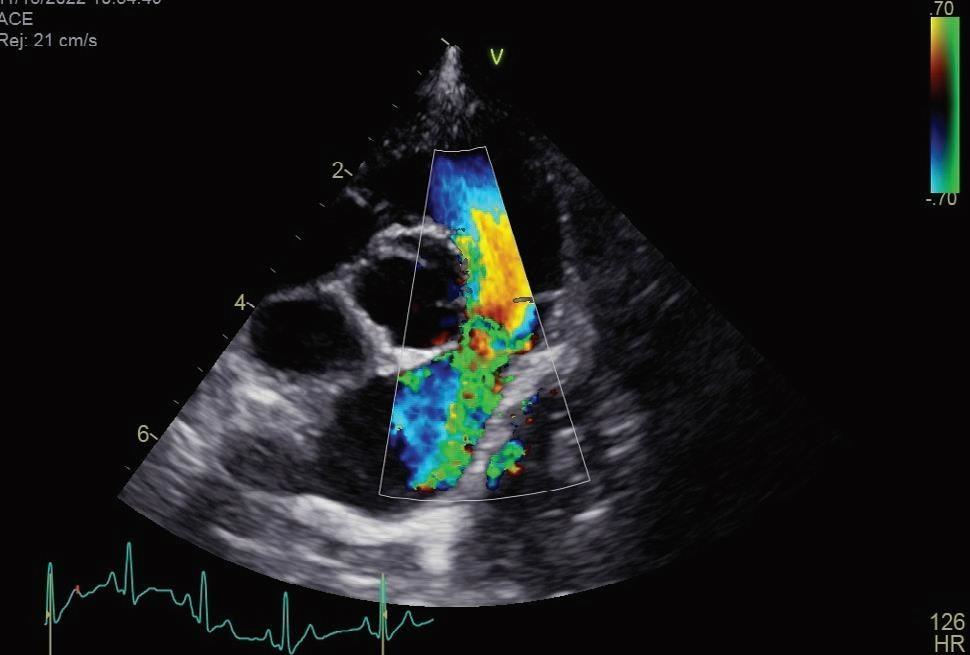
(b)

**Figure:**
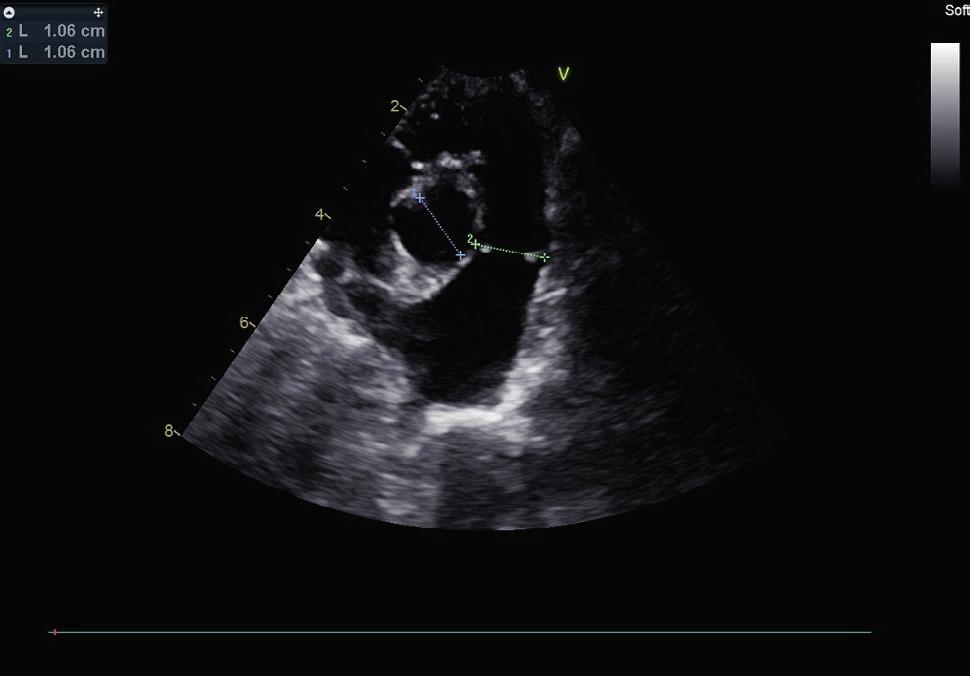
(c)

**Figure:**
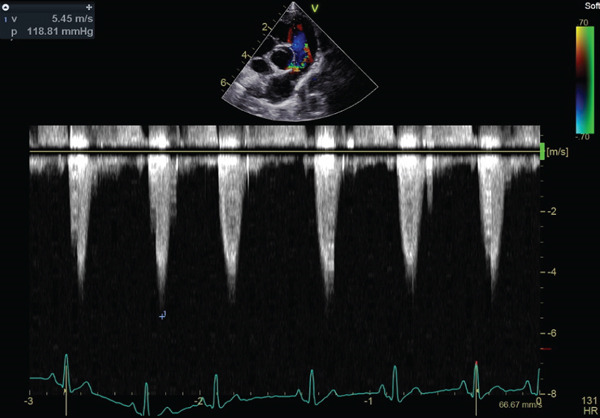
(d)

A hypoechoic vascular structure was observed at the lateral position adjacent to the left atrium in the area where the coronary sinus is located (Figure 2a,b and Video S2). On the basis of the echocardiographic findings, the dog was diagnosed with severe PS and PLCVC. Based on the history, size, and diagnostic findings, right ventricular outflow tract reconstruction under extracorporeal circulation was recommended rather than percutaneous balloon valvuloplasty. After obtaining the owner′s consent, the surgical procedure was performed 31 days after the first visit.

Figure 2Echocardiographic images of right parasternal long‐axis views of the heart on initial examination. (a, b) Echocardiography suggested that the PLCVC coursed along the left atrium and ultimately drained into the right atrium.

**Figure:**
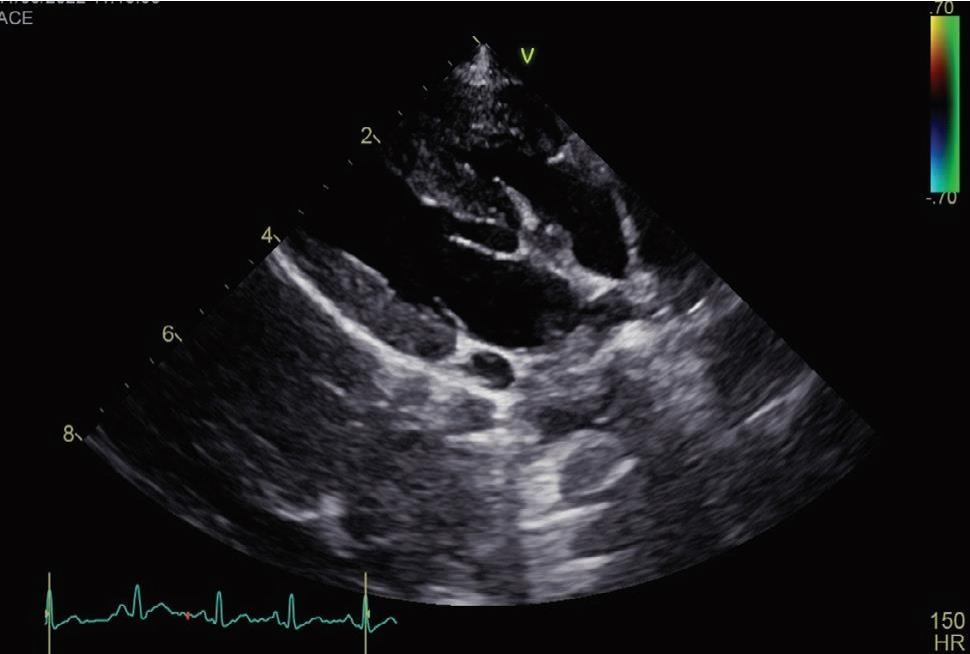
(a)

**Figure:**
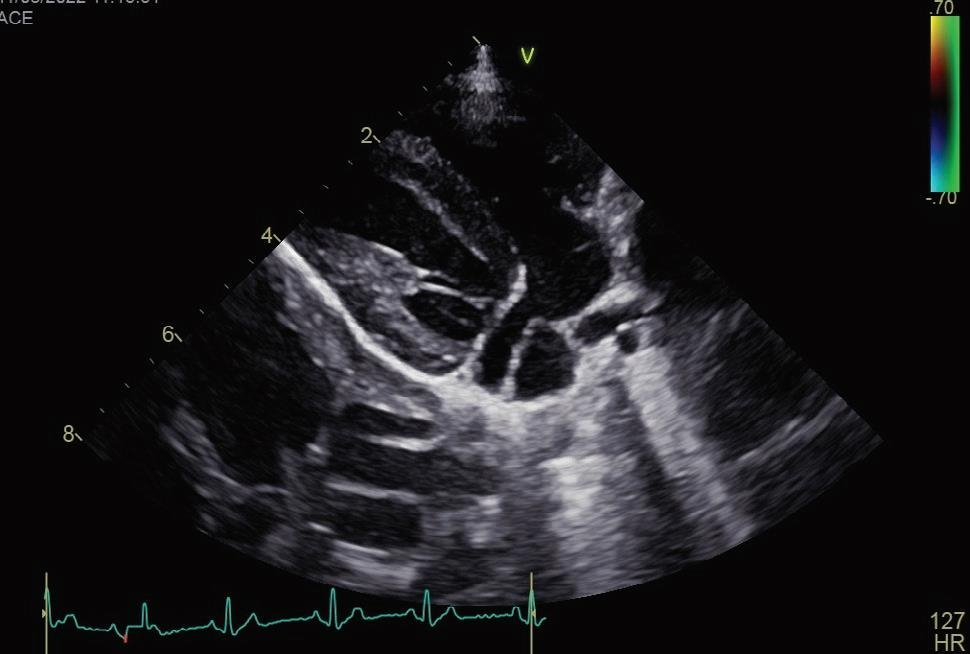
(b)

Preanesthetic medications consisted of midazolam (0.3 mg/kg intravenously [IV]), fentanyl (5 *μ*g/kg IV), propofol (2 mg/kg IV), and cefazolin sodium (25 mg/kg IV). The dog was preoxygenated (100% O_2_), induced with propofol (to effect IV), and intubated with an endotracheal tube. Anesthesia was maintained with 1.5%–3% sevoflurane in 100% O_2_ at 1.5 L/min. Heart rate, respiratory rate, rectal temperature, esophageal temperature, arterial oxygen saturation, end‐tidal CO_2_, and sevoflurane concentration were monitored continuously using a biological monitor. The dorsalis pedis artery was catheterized (24G indwelling cannula) to obtain systolic, diastolic, and mean arterial pressure measurements. Packed cell volume, hematocrit, total protein concentration, activated clotting time, and arterial blood gases were determined as necessary, using blood samples obtained from the dorsalis pedis artery catheter. A 5‐Fr polyethylene catheter was passed into the bladder and used to measure urine output.

In preparation for CPB, the left carotid artery and jugular vein were surgically isolated. A left thoracotomy was performed at the fourth intercostal space after an intercostal nerve block with bupivacaine hydrochloride (Figure 3a,b).

Figure 3Intraoperative images. (a) Appearance of the heart immediately after thoracotomy. The persistent left cranial vena cava (PLCVC) was observed directly above the pulmonary artery and left atrial appendage. (b) Appearance of the heart after making a pericardial basket. Dilatation of the pulmonary trunk was observed.

**Figure:**
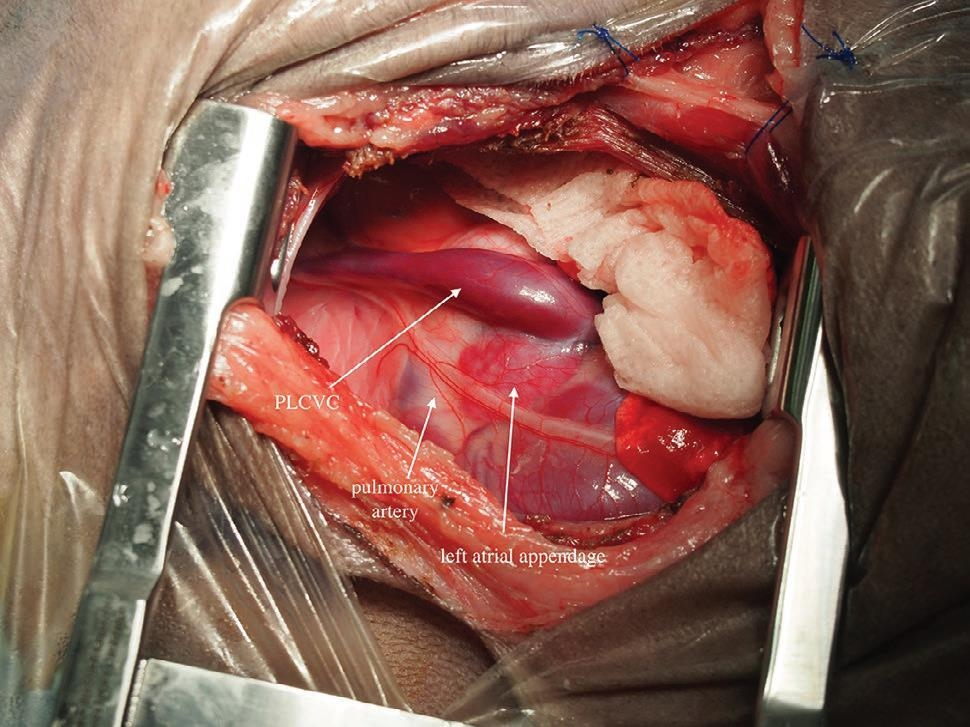
(a)

**Figure:**
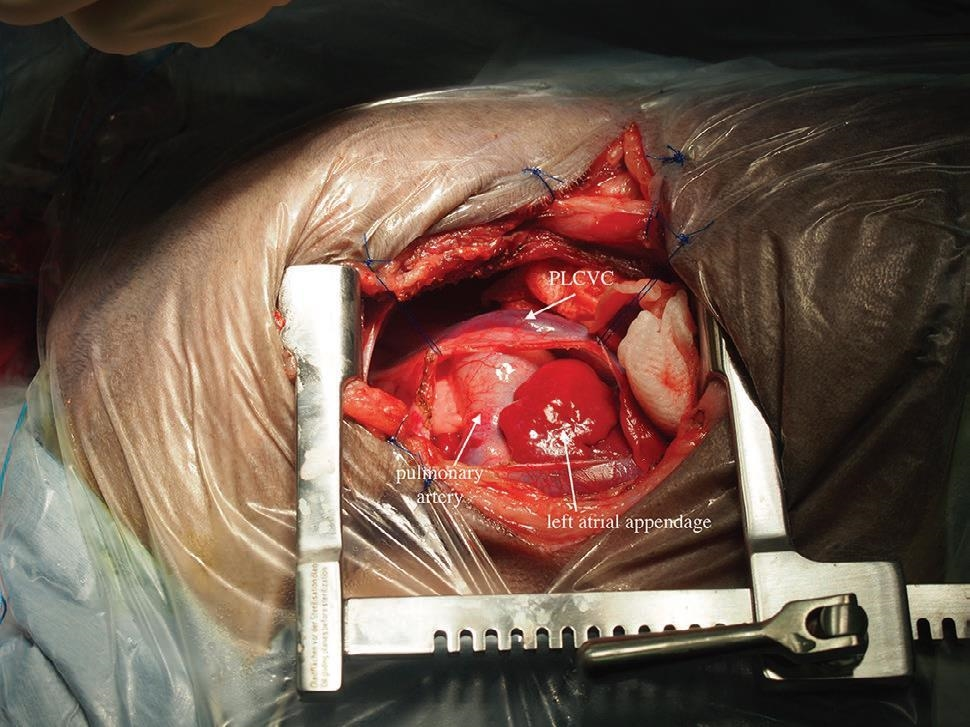
(b)

Subsequently, heparin sodium (200 U/kg IV) was administered to the dog in order to achieve an activated clotting time of > 400 s. After 3 min, the activated clotting time was measured and confirmed to be > 400 s. A 6‐Fr CPB cannula was inserted into the carotid artery as the arterial line of the CPB. For venous return, a 10‐Fr CPB cannula was inserted into the jugular vein.

Cardiopulmonary bypass was established using a heart–lung machine with an extracorporeal circuit, a 0.6‐m^2^ oxygenator, and a heat exchanger. The CPB circuit was primed with 20% D‐mannitol (5 mL/kg), heparin sodium (100 U/kg), furosemide 5 mg, and 7% sodium bicarbonate (150 mL). After the air was removed from the CPB circuit and activated clotting time was further prolonged (1000 s), a partial CPB was initiated, and the dog′s body temperature dropped to 28°C. Blood flow was set at 60–100 mL/kg/min on the CPB pump.

Under total perfusion of the extracorporeal circulation, the pulmonary artery was incised longitudinally with a scalpel. Subsequently, a partial valvectomy was performed by excising approximately 3–4 mm from the free edge of the thickened pulmonary valve leaflets to reduce leaflet redundancy and improve valve opening. Commissurotomy was then performed, with the incisions extended to near the annulus to enlarge the valvular orifice. Blood escaping from the pulmonary artery was retrieved by suction and returned to the cardiopulmonary bypass system (Video S3). Although ventricular fibrillation occurred during valve resection, hemodynamics remained stable on CPB. Two simple continuous sutures were then placed at the arteriotomy site. After completion of pulmonary arteriotomy closure, a single electrical defibrillation shock (0.5 J; 0.2 J/kg) restored sinus rhythm while the patient remained on CPB. The interval from the first incision to the completion of suturing of the pulmonary artery was about 51 min.

After ensuring stable hemodynamics, the dog was weaned from the extracorporeal circulation, and the venous return catheters were removed. To antagonize the effect of heparin, 3 mg/kg of protamine sulfate was administered IV over 30 min. The chest was closed after a drain tube was placed using standard methods. The lowest esophageal temperature was 26.0°C. The durations of extracorporeal circulation and anesthesia were 111 min, and 330 min, respectively. The dog recovered uneventfully from anesthesia. After extubation, the dog was observed in the intensive care unit for 12 h and then transferred to general care. Postoperatively, an antibiotic (cefazolin 25 mg/kg IV twice daily for 7 days) and analgesic (robenacoxib 2 mg/kg subcutaneously once daily for 7 days) were administered. The dog recovered well without complications, and the cardiac murmur decreased to Grade II/VI. The dog was discharged on the 7th day after surgery and was prescribed clopidogrel (2 mg/kg orally once daily for 3 weeks).

At 1 month postoperatively, the dog showed no signs of exercise intolerance, and Doppler echocardiography revealed a reduced pulmonary artery velocity of 2.30 m/s (pressure gradient of 21.16 mmHg) (Figure 4a,b and Video S4). PV mean gradient was 5.38 mmHg, and the VTI ratio was 0.414 (VTI_AV, 10.5 cm; VTI_PV, 25.39 cm). At the 6‐month follow‐up, PV max was 1.98 m/s (pressure gradient of 15.68 mmHg), PV mean gradient was 4.49 mmHg, and the VTI ratio was 0.438 (VTI_AV, 9.83 cm; VTI_PV, 22.46 cm), with no echocardiographic evidence of clinically relevant restenosis.

Figure 4Echocardiographic images 1 month after surgery. (a, b) Echocardiography showed no thickened pulmonary valve. The systolic blood flow velocity across the right ventricular outflow tract is 2.30 m/s, and the pressure gradient is 21 mmHg according to Bernoulli equation.

**Figure:**
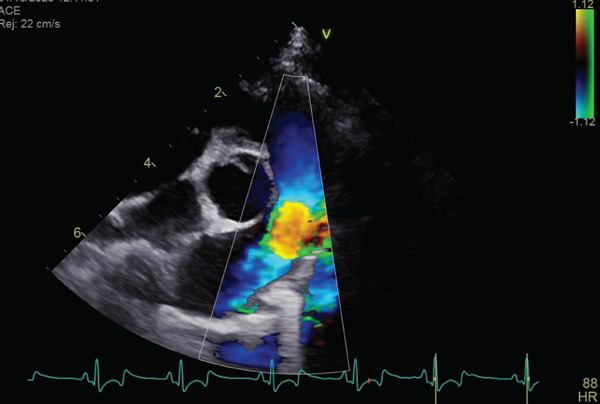
(a)

**Figure:**
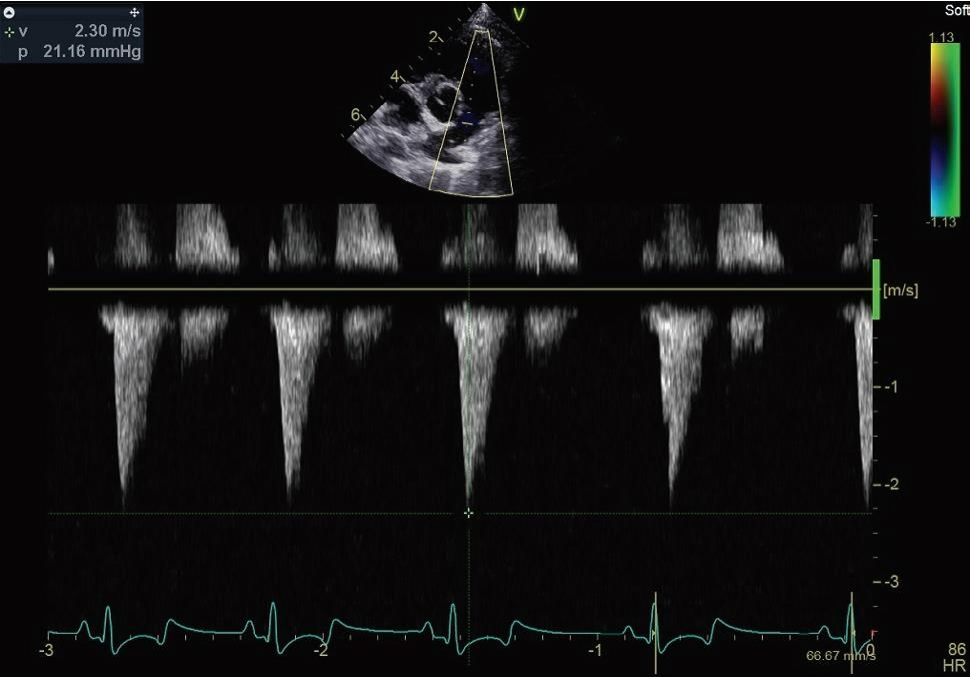
(b)

## 3. Discussion

In this dog, surgical correction under cardiopulmonary bypass was selected because catheter‐based intervention was considered technically challenging due to the patient′s small body size and the presence of PLCVC [10, 11, 13]. To the best of our knowledge, this is the first report describing successful surgical correction of PS complicated by PLCVC using extracorporeal circulation in a dog. Beating‐heart cardiopulmonary bypass was chosen to avoid myocardial ischemia and to reduce the complexity of myocardial protection in a small patient. Although ventricular fibrillation occurred intraoperatively and was intentionally tolerated until completion of pulmonary arteriotomy closure to facilitate suturing, the primary rationale for using beating‐heart CPB in this small patient was to avoid aortic cross‐clamping and cardioplegic arrest, thereby preventing planned global myocardial ischemia and simplifying myocardial protection. During this fibrillatory period, systemic perfusion pressure and oxygen delivery were maintained by full‐flow CPB under mild hypothermia, and sinus rhythm was restored by electrical defibrillation before weaning from CPB. Nevertheless, we acknowledge that prolonged ventricular fibrillation may attenuate the benefits of an on‐beating strategy, and early rhythm restoration or conversion to an arrested‐heart technique should be considered if hemodynamic stability or myocardial protection becomes a concern. From a technical standpoint, visualization of the pulmonary artery incision line was impaired by the presence of PLCVC. In cases in which excessive vascular curvature is present, insertion of a venous return cannula into the PLCVC may be technically challenging. In the present case, the venous return cannula was inserted before establishment of the pericardial cradle to facilitate access. Severe PS likely increased right‐sided filling pressures and may have contributed to dilation of the PLCVC. However, because angiographic assessment (CT or fluoroscopy) of the right cranial vena cava was not performed, alternative venous anatomy (e.g., absent/hypoplastic right cranial vena cava with redistribution of cranial venous return) cannot be excluded. Regardless of the underlying cause, the enlarged caliber of the PLCVC in this dog facilitated identification of the vessel and manual guidance of the cannula from the cervical region into the thoracic cavity. However, care must be taken to avoid forceful advancement when resistance is encountered, as the thin vascular wall may predispose to vessel rupture. This case was classified as type A pulmonary valve stenosis based on preoperative echocardiographic findings. Although balloon valvuloplasty is widely used for the treatment of PS because it is less invasive and generally safer than open‐chest surgery, technical limitations have been reported, particularly in small dogs due to their body size and vascular anatomalies, such as PLCVC in the present case, may complicate catheter manipulation and positioning across the stenotic pulmonic valve. [5, 14]. Bilateral peripheral venous contrast echocardiography (e.g., injections from both cephalic veins) may help delineate cranial venous anatomy and drainage patterns, and may assist in determining whether a right jugular approach for balloon pulmonary valvuloplasty is feasible (i.e., whether a right cranial vena cava drains directly into the right atrium). Although this was not performed in the present case, it may be considered in similar patients. In the present case, however, balloon pulmonary valvuloplasty was considered less suitable due to the patient′s very small body size and the anticipated complexity/uncertainty of cranial venous anatomy; therefore, surgical correction under CPB was selected. The cardiopulmonary bypass approach used in this case, combining a cervical access route with left thoracotomy, has been increasingly reported in small animal cardiac surgery [15, 16]. Typically, venous drainage is achieved via the jugular vein to the right atrium. When venous access is limited by abnormal anatomy, such as PLCVC, additional venous drainage via the right atrial appendage or alternative surgical approaches, including median sternotomy, may be considered. In the present case, adequate pump flow was achieved using jugular venous drainage alone, and additional cannulation was not required. Catheterization from the jugular vein to the right heart can also be challenging in dogs with PLCVC. In dogs, PLCVC most commonly coexists with a right cranial vena cava (resulting in bilateral cranial vena cavae); however, cases with absence of the right cranial vena cava have also been reported, in which venous return from the head and neck and forelimbs drains predominantly via the PLCVC (through the coronary sinus) [17, 18]. Accordingly, venous return via the PLCVC passes through the coronary sinus before entering the right atrium, potentially complicating catheter navigation. Preoperative angiography or computed tomography may therefore be useful for surgical planning and selection of an optimal approach.

This report is limited by the description of a single case; therefore, further studies involving additional cases are required to evaluate the long‐term outcomes and reproducibility of this approach. Despite the potential risk of bleeding associated with surgical intervention compared with balloon valvuloplasty, the procedure was performed safely in this dog. In conclusion, surgical correction under cardiopulmonary bypass represents a viable treatment option for dogs with severe PS complicated by PLCVC.

## Funding

No funding was received for this manuscript.

## Consent

No written consent has been obtained from the patient, as there is no patient‐identifiable data included in this case report.

## Conflicts of Interest

The authors declare no conflicts of interest.

## Supporting Information

Additional supporting information can be found online in the Supporting Information section.

## Supporting information


**Supporting Information 1** Video S1: Echocardiographic examination of right parasternal short‐axis views of the heart moderate right ventricular hypertrophy and type A valvular pulmonary stenosis.

**Supporting Information 2** Video S2: Echocardiographic examination of long‐axis views. PLCVC draining into the right atrium.

**Supporting Information 3** Video S3: Compilation of intraoperation images of case during right ventricular outflow tract reconstruction.

**Supporting Information 4** Video S4: Echocardiographic examination of right parasternal short‐axis views after surgery.

## Data Availability

The data that supports the findings of this study are available from the corresponding author upon reasonable request.
